# What is a good health check? An interview study of health check providers’ views and practices

**DOI:** 10.1186/s12910-017-0213-x

**Published:** 2017-10-02

**Authors:** Yrrah H. Stol, Eva C. A. Asscher, Maartje H. N. Schermer

**Affiliations:** Department of Medical Ethics and Philosophy, ErasmusMC, Na building, room Na 21.197 Postbus, 2040 3000 CA Rotterdam, The Netherlands

**Keywords:** Health check, Screening, Ethics, Criteria, Interviews, Qualitative research, Informed consent

## Abstract

**Background:**

Health checks identify (risk factors for) disease in people without symptoms. They may be offered by the government through population screenings and by other providers to individual users as ‘personal health checks’. Health check providers’ perspective of ‘good’ health checks may further the debate on the ethical evaluation and possible regulation of these personal health checks.

**Methods:**

In 2015, we interviewed twenty Dutch health check providers on criteria for ‘good’ health checks, and the role these criteria play in their practices.

**Results:**

Providers unanimously formulate a number of minimal criteria: Checks must focus on (risk factors for) treatable/preventable disease; Tests must be reliable and clinically valid; Participation must be informed and voluntary; Checks should provide more benefits than harms; Governmental screenings should be cost-effective. Aspirational criteria mentioned were: Follow-up care should be provided; Providers should be skilled and experienced professionals that put the benefit of (potential) users first; Providers should take time and attention. Some criteria were contested: People should be free to test on any (risk factor for) disease; Health checks should only be performed in people at high risk for disease that are likely to implement health advice; Follow up care of privately funded tests should not drain on collective resources.

Providers do not always fulfil their own criteria. Their reasons reveal conflicts between criteria, conflicts between criteria and other ethical values, and point to components in the (Dutch) organisation of health care that hinder an ethical provision of health checks. Moreover, providers consider informed consent a criterion that is hard to establish in practice.

**Conclusions:**

According to providers, personal health checks should meet the same criteria as population screenings, with the exception of cost-effectiveness. Providers do not always fulfil their own criteria. Results indicate that in thinking about the ethics of health checks potential conflicts between criteria and underlying values should be explicated, guidance in weighing of criteria should be provided and the larger context should be taken into account: other actors than providers need to take up responsibility, and ideally benefits and harms of health checks should be weighed against other measures targeting (risk factors for) disease.

**Electronic supplementary material:**

The online version of this article (10.1186/s12910-017-0213-x) contains supplementary material, which is available to authorized users.

## Background

### Population screening and personal health checks: Benefits and harms

Health checks identify (risk factors for) disease.^1^ Unlike diagnostic tests, they are offered to or requested by people without specific symptoms. [1] Health checks can also be referred to as preventive or presymptomatic tests, medical screening or preventive medical examinations. For ease of reading, ‘health check’, ‘check’ and ‘test’ are used interchangeably throughout this paper.

Health checks may be offered by different providers. If provided by the government we refer to them as population screening programs. In the Netherlands, there are population screening programs for breast cancer, bowel cancer and cervical cancer. In the United Kingdom, there is also screening for risk factors for cardiovascular disease (CVD). Typically, people in the target group receive an invitation to participate. Most population screening programs are provided free of charge.

Health checks may also be provided by other parties, and this is increasingly the case. In the Netherlands, physiotherapists, pharmacies, General Practitioners (GPs), gyms, Non Governmental Organisations such as the Heart Foundation, specialized medical centres and companies offer checks on a wide variety of (risk factors for) disease, including cancer and CVD. Examples include the total body scan, tests for prostate specific antigen (PSA) and genetic tests. Because these tests are not offered to (sub)populations but to individuals – through advertisements for example – we will refer to them as ‘personal health checks’ throughout this paper. Note that the distinction between population screenings and personal health checks depends on whether the offer is directed at a population or at an individual. The same test can be used in population screening programs and as personal health check.

Population screening programs and personal health checks can provide insight into one’s health status. When risk factors or latent disease are uncovered, preventive action may improve health. However, not all conditions are treatable. In addition, false positive or negative results may lead to unnecessary treatment or unjustified reassurance with potentially negative consequences for health. If the course of disease is hard to predict even accurate diagnosis may lead to overtreatment. Some tests carry risks in themselves, such as invasive tests or tests using radiation. [2] Moreover, health check offers may indirectly result in decreased health solidarity and unfairly distributed health outcomes. [3] The weighing of benefits and harms for individuals and society requires ethical evaluation. [1–3]

Ethicists usually do not distinguish between population screening and personal health checks when it comes to benefits and harms and the weighing thereof. [2, 4–6] This is different however in policy and regulation.

### Evaluation of population screening and personal health checks in policy, law and regulation

Traditionally, policymakers and public health professionals use ethical criteria for responsible screening, derived from the Wilson and Jungner criteria, to evaluate whether or not population-screening programs should be offered. [7–10] Criteria for responsible population screening state that the screening should have clinical utility^1^, the benefits for the participants must outweigh the drawbacks, the test method must be both analytically valid^2^ and clinically valid,^3^ participation must be voluntary and based on reliable information, the offer and performance of screening should be in accordance with patient- and consumer rights, and the screening must be responsible in terms of the use it makes of public and collective health service resources. [summary derived from 5] There is discussion about whether or not specific population screening programs meet these criteria, but the criteria themselves are relatively uncontested. [9] In the Netherlands and Belgium there is also legal evaluation of population screening. [11, 12] In the Netherlands this concerns a mandatory permit for screening that uses ionizing radiation, aims to detect cancer or diseases for which there is no treatment or prevention. Grounds to refuse a permit are (i) the screening test is scientifically unsound; (ii) the screening does not conform the legal rules for medical treatment; (iii) the expected benefit of the screening does not outweigh the risks for the participants. To determine the risk–benefit ratio for participants, the Wilson and Jungner criteria are used. [9]

As stated, most ethicists think that the Wilson and Jungner criteria are also applicable to personal health checks. [2, 4–6] However, policymakers disagree on the question whether personal health checks – like population screening – should be regulated and if so, which criteria should be used. For the ethical evaluation of personal health checks, several policy proposals have been made.^4^ On a European level quality criteria have been developed [13], in the United Kingdom, principles for direct to consumer genetic tests have been designed that may be applied to all personal health checks across all jurisdictions [14], the Health Council of the Netherlands (HCN) has written on the ethical evaluation of personal health checks [1] and the Royal Dutch Medical Association has published a guideline. [15] The latter has been withdrawn but was in operation during the time of this study. In the Netherlands – as in other European countries – there is limited regulation of personal health checks. [11] Several parties have indicated that more regulation, or more compliance with and oversight of existing laws and regulations, is necessary to protect citizens against harmful effects of personal health checks. [1, 12]

As yet, there is no consensus on the criteria for ‘good’ personal health checks. The criteria in existing policy proposals vary, but are less stringent than criteria for responsible population screening: For example, none of the published guidelines mentions clinical utility and cost-effectiveness. [1, 13–15]. Clinical validity is only considered a criterion by the HCN [1], analytic validity by the HCN [1] and HGC. [14] Just as criteria for responsible populations screening, criteria for personal health checks mention informed consent. [1, 13–15] The question why criteria for personal health checks divert from criteria for responsible population screening, is answered by the Health Council of the Netherlands stating that ‘health gains are not necessarily a precondition for the individual. For the individual, the results obtained may be of use purely as information or they may be valuable in terms of reassurance. Also, cost-effectiveness is not a prime concern for the individual.’ [1]

### Towards a new ethical framework for personal health checks

There is currently much public, policy and political debate in the Netherlands on the regulation of health checks. [16–19] This study aims to contribute to an ethical framework for responsible offers and use of personal health checks. We aim to formulate context-sensitive yet general criteria that – like the Wilson and Jungner criteria – may be applied to all sorts of tests. Criteria may also help providers in evaluating and adjusting their health check offers.

In order to develop ethical criteria for personal health checks that may be used to inform policy, the experiences and perspective of all stakeholders should be considered as they may identify different values, ethical dilemmas and practical barriers. Here we describe the results of an interview-study of providers’ views on criteria for good health checks. The perspective of health check providers is of particular importance, because they – within the limits of the law – determine which checks are offered to whom in which way. First, if their perspective differs in important aspects from criteria, a nonbinding guideline may not suffice to improve the offer of health checks. In fact, this is one of the reasons that the aforementioned guideline of the Royal Dutch Medical Association has been withdrawn: its effects were limited because commercial providers of health checks did not endorse it. [20] Second, providers are well positioned to identify practical and ethical barriers in the implementation of ethical (criteria for) personal health checks. Some of these may be removed.

In our interview-study with a wide range of providers, we investigate what they consider criteria for ‘good’ health checks, whether they differentiate between criteria for population screening and for personal health checks, and we explore what role the aforementioned criteria play in their practices.

Although providers have been involved in the development of earlier criteria and guidelines [1, 13–15] and have been interviewed about the desirability of particular health checks in evaluation studies (e.g. [21, 22]), this is – as far as we know – the first paper on the views of a wide range of providers on general criteria for good health checks (i.e.preventive or presymptomatic tests) would be.

Of course, the criteria as formulated by providers should not be taken as a final say, but used as input in the ethical and policy debates over the right criteria. We therefore also provide an ethical evaluation of providers’ criteria and practice.

## Methods

### Interviews

Twenty semi-structured interviews were conducted in Dutch by Yrrah Stol (YS) in 2015 lasting 45–90 min. With one exception, interviews were conducted at the workplace of providers of health checks. Respondents were asked to share their opinions freely and were assured their answers would be anonymized. They all consented to publication of findings. We asked them what kind of check(s) they offer, their reasons for doing so, and the benefits and potential drawbacks of these checks. Subsequently they were asked an example of a good personal health check, and what general criteria for ‘good’ personal health checks would be. Similar questions were raised about ‘bad’ checks. Although we did define health checks (‘checks on (risk factors for) disease in people without specific medical indication’) we deliberately left open the question of what aspects of a ‘good’ or ‘bad’ check would be so that respondents were free to mention test- and disease characteristics, but also features of the provider, offer or context in which this offer is made. Providers were also asked what they would consider desirable policy concerning personal health checks. Finally, we asked them whether their current practice leaves room for improvement and how this may be achieved. (Additional file 1) Before the start of the interview, we told providers interviews would be used to inform an ethical framework for health checks that we are developing to guide policy.

### Participants

Interviews were conducted with direct providers as well as managers of health check businesses: GPs (GP), physiotherapists (PHY), health and safety officers (HSO), pharmacists (PHA), population screening program officers (PSPO), screening by GP program officers (GPSPO), independent treatment centres providing insured (ITC-I) and uninsured (ITC-U) care, and Internet based companies (IC). (Additional file 2) They offered the following health checks to the Dutch public: tests for cardiovascular risk factors (sugar, blood pressure, cholesterol, BMI, questionnaire on lifestyle), tests for cancer (PSA test, rectal examination, mammography, cervical smear, stool test), total body scans, and other blood tests (e.g. sexually transmittable diseases (STD) tests, thyroid- kidney- and liver function, vitamin-, mineral- and hormone tests, hepatitis B&C tests).

We chose the range of providers based on our own knowledge of the field. We included both providers of all commonly performed checks (such as cholesterol tests and governmental screenings) and providers of the checks that are currently widely discussed in the Netherlands (such as the total body scan and PSA tests). Although they themselves do not provide personal health checks, governmental screening officers were approached because of their knowledge of the field. Our aim was to include a sample of health check providers that was as diverse as possible because different providers may stress different criteria. We interviewed medical specialists in commercial and regular care,^5^ directors and key players of big organisations as well as health check providers in one-man companies, providers that conduct health checks face to face as well as those who supply via Internet. Sex and age of providers were no selection criteria, as we did not consider it likely that these characteristics would influence criteria. We identified individual candidates from our own network, through an internet-search, via other experts on health checks in the Netherlands and via snowballing. Providers were approached by e-mail and then called by telephone to ask whether they were interested to be interviewed. Of the twenty-eight providers that we approached, twenty agreed to an interview. We managed to arrange interviews with major players in the field. However, we did not succeed in including ‘small’ commercial providers without a (para)medical background: five of the eight providers that declined were small commercial providers.

Saturation as regards to criteria for health checks was reached long before the last interview.

### Analysis

Interviews were recorded and transcribed verbatim. They were coded using NVIVO 10.2.2 for Mac focusing on characteristics of ‘good’ and ‘bad’ tests. An initial list of bottom-up derived codes reflecting characteristics of good and bad health checks and a range of other themes (such as information, autonomy and the organization of healthcare) was composed and discussed between YS and Eva Asscher (EA) after 10 interviews. Additional bottom-up codes were added during the analysis, staying close to the content of the answers of respondents. Similar codes were merged. Two interviews were double-coded by EA to ensure agreement about the codes, there was agreement about the codes used. Analysis of codes to results was done by YS in close consultation with EA and Maartje Schermer (MS). Quotes were translated from Dutch to English by YS.

As we concentrate on criteria for good health checks in this paper, we report on provider’s reasons for offering checks and their opinion on policy only when relevant.

## Results

### Criteria for good personal health checks

Providers consider characteristics of the test itself, the disease tested on and how health checks are performed (the offer, provider and target population) part of what makes a test good or bad.

We divided the criteria mentioned by providers in three categories: Minimal criteria were (almost) unanimously mentioned by interviewed providers. These criteria are moreover decisive in the distinction between good and bad test: respondents described tests that do not meet minimal criteria ‘bad’. E.g. a good test is reliable and valid, a bad test unreliable and invalid. Aspirational criteria were mentioned by only part of the providers. Checks that do not meet these criteria are not necessarily considered ‘bad’, respondents merely think that health check offers could be improved by complying with these criteria. Contested criteria are aspirational criteria where providers (heavily) disagree about.

We discuss all types of criteria and illustrate them with quotes. The selection of quotes is based on their conciseness and appeal, and represent different providers. For reasons of anonymity, we may leave aside whether providers refer to their own health checks or the offer of others.

Criteria are summarized in Table 1.

**Table 1:** Minimal, aspirational and contested criteria for good personal health checks as formulated by interviewed providers

Minimal criteria	Treatability of (risk factors for) disease: Health check results must provide clear opportunities for health improvement
	Clinical validity: Health check results must be valid and reliable indicators for (risk factors for) disease
	Informed consent: Participation must be voluntary and based on reliable information
	Health checks should provide more benefits than harms
	In case of population screenings: cost-effectiveness
Aspirational criteria	Follow-up care should be provided, including objective explanation of test-results and facilitation in the realization of health benefits
	Providers should be skilled and experienced professionals that put the benefit of (potential) users first
	Providers should take time and attention
Contested criteria	People should be free to test on any (risk factor for) disease, provided they are well-informed
	Assessment before the test: Health checks should only be performed in people at high risk for disease
	Follow up care of privately funded tests should not drain on collective resources

#### Minimal criteria

1. *Health check results must provide clear opportunities for health improvement*

To benefit users, nearly all providers state that a good check must provide clear opportunities for health improvement:

First and foremost, you should perform a test only in those cases in which you can act on the results, the condition should be treatable, it should be possible to relieve symptoms [once they present themselves YS]. But preferably that you can detect it in such an early stage that it can be prevented. That, for me, is the main characteristic of a good test (HSO).

In the end, it has to be a treatable condition. (GP).

(Risk factors for) disease must be treatable either through preventive lifestyle changes, medical intervention or changes in the environment. Moreover, providers almost unanimously characterise health checks that (also) test for diseases for which no treatment or prevention is available as ‘bad’; these would harm users by causing concern and a loss of experienced health:

You have to be able to act on it. You need to give a therapy option. If you don’t have one (…) one becomes worried and therefore it is not a good test (ITC-U).

The total body scan^6^ is an often-mentioned example of a test that may reveal untreatable conditions.

2. *The health check must be reliable and valid*

The second criterion providers almost unanimously mention is that personal health checks must be reliable and valid tests. Providers stress both the importance of analytical and clinical validity: If the conditions remain the same, results should be consistent, and personal health checks should accurately predict the presence or absence of the (risk factor for) disease. This is operationalized in a high sensitivity and specificity and good positive and negative predictive value for the (risk factor for) disease:

A reliable, valid test, that is [criterion] one (PHA).

The higher the sensitivity, the higher the specificity, the better it is (ITC-U).

It is remarkable how many providers stress the importance of combining tests for risk factors: this would give a better indication of disease developing in the future. According to providers, risk factors for cardiovascular disease (CVD) should be considered in combination, and Prostate Specific Antigen (PSA) should be checked in combination with a rectal examination:

Cholesterol alone is not enough. For only a part of a cardiovascular risk profile, but that’s it. (…) If you want to compose a cardiovascular risk profile.. Then I would say yes that makes sense (HSO).

80% of the men referred (…) [to this clinic] with an increased PSA did not get a rectal examination. The first step in diagnosing prostate cancer.. 80% did not get a rectal examination (ITC-I).

Checks that do not meet the reliability and validity criterion are characterized as ‘bad’ by nearly all providers because they may harm individuals. People should not be falsely reassured and false positive test results should be kept to a minimum because they may cause medicalization, unnecessary worries, over-treatment and unnecessary burdens on the health care system:

R: Sensitivity and specificity are truly essential. (…) I think that there are many health checks in which the tests are such that they result in a substantial amount of false positives. I think yes, many worried people will be referred with all the associated consequences. I: What consequences do you have in mind? R: overdiagnosis to start with, more referrals to health care, perhaps burdensome examinations, a trajectory full of uncertainty (PSPO).

A test on total-cholesterol is an often-mentioned example:

It tells you nothing, total cholesterol. It can be five or four, you think great, but it could be that the [LDL/HDL] relation is skewed, so these people are left none the wiser: you’re okay. I consider that even worse (ITC-U).

3. *Participation must be voluntary and based on reliable information*

Providers consider it problematic when people feel pressured or are actually pressured to test due to the conflict with self-determination:

Some people want to know, and others do not at all. People should be free in this decision. One certainly should have the opportunity to say ‘No, I prefer not to know’ (GPSPO).

Good information is almost unanimously mentioned as characteristic of a good check. Potential users should be informed about the advantages, disadvantages and limitations of the check beforehand to ensure informed consent (or dissent):

I consider that one of the most important criteria for good health checks, and that is that you provide people with sufficient and reliable information in advance (ITC-U).

Good information is very important, according to me. And good information may also result in a decision not to test (HSO).

Some providers also explicitly state they consider a check ‘bad’ when informed consent is lacking.

4. *Health checks should provide more benefits than harms for the individual*

Some providers explicitly mention this overarching criterion:

The balance between pros and cons, that is crucial to me (PSPO).

There are two sides to each check. (…) You aim for something that people.. which is quite specific .. where people can act upon, where they can do something about. (...) But the downside, the risk is always that it leads to medicalization (HSO).

Many state that health checks should not cause direct harm and only minimal burden. In fact, they almost unanimously state that the government should forbid the offer of directly harmful tests (eg using X-rays)^7^ or should ensure these tests meet high quality standards:

People need to be protected against unsound or really dangerous tests. So it should be possible to ban unsound or dangerous health checks (ITC-U).

Often this criterion refers not only to the test itself, but also to the whole trajectory of testing and treatment. Many providers express worries concerning over-treatment of prostate cancer which may cause impotence and incontinence while the detected cancer may not have resulted in health problems:

It results in a nasty urological treatment, that could be useful for some, but is not for the majority (GP).

To assure health benefits and to avoid harm, the presence of disease should have implications for (future) wellbeing.

In case of governmental screening programs, interviewed providers formulate an additional minimal criterion:

5. *Health checks should be cost-effective*

Providers differentiate between personal health checks and governmental screening programs when it comes to the cost-effectiveness of the check. Governmental screening programs should be cost-effective: the costs of the screening program should be in proportion with the expected gain in health.

Costs certainly play a role in governmental screening programs. The tests should be affordable. (PSPO).

An individual check up is very different to population screening in this regard. For the moment you’d say ‘everybody between age fifty and seventy gets a [free YS] total body scan’, you’ll make costs of formidable size (ITC-U).

### Aspirational criteria

1. *Follow-up care should be provided, including objective explanation of test-results, referral to specialized care and assistance with the realization of health benefits*

Many providers stress that results are meaningless unless properly interpreted and put in perspective. Explanation of results may also prevent harm resulting from overtreatment and unnecessary worries. An often-mentioned example in this regard is the PSA test. Providers should take sufficient time to explain that prostate cancer need not always to be treated but may in some instances better be monitored with regular rectal examinations, PSA tests, biopts and/or MRI’s.

Interviewed providers also stress that users need to be referred to specialized care if tests results warrant so.

Finally, to realize health benefits after the test, users should be informed about, advised on, and – according to some providers – if necessary assisted with the realization of treatments and/or lifestyle adjustments:

If a doctor says “go exercise more”, that is one thing, but to actually do it is something altogether different. And I often think that many people really need a big stick and need some advice. It is often easier said than done is my experience. (Physiotherapist).

2. *Providers should be skilled and experienced professionals that put the benefit of (potential) users first*

Many providers think that health checks should preferably be offered and performed by skilled and experienced health care professionals because they have the capabilities to properly inform (potential) users about the benefits and harms of a test, to interpret test results and to explain their relevance to users:

Quality has to do with the quality of the test, and with the quality of the doctor. A test may have a high sensitivity, high specificity, but if the doctor is not able to interpret the results … I always stress, I find it very important that people realize that early diagnosis is a profession. In everybody I investigate I’ll find something. Of course, because I’m looking at a body and no one body is built the same, so you’ll always find variations on the norm. And it’s very important to get to know those variations on the norm so you know when to act and when not to do so. (ITC-U).

Furthermore, some providers state that the interests of (potential) users should come first in the provision of health checks. They warn for (financial) incentives to test or treat in commercial providers, but also in regular care:

When it comes to prostate cancer, the biggest crime is the robots that those hospital directors have put down. To their urologists, they say ‘I’ll buy a robot for you if you remove 200 prostates’. That’s horrible. (...) Don’t do a [PSA] test at a urologists under pressure to operate (ITC-I).

3. *Providers should take time and attention*

In order to properly inform, advise and assist (potential) users before and after the test, providers emphasise the importance of time and empathy:

You need to take the time to explain. (…) Time, time.. That is of crucial importance to patients (ITC-I).

### Contested criteria

1. *People should be free to test on any (risk factor for) disease, provided they are well-informed*

A large number of the providers, including all commercial ones, state that testing or not should ideally be an individual choice. With the exception of directly harmful health checks, people should be able to do a personal health check on any (risk factor for) disease, provided they are well-informed:

Health is our most precious possession, and every individual, every citizen has the right to make decisions about their own health. And thus to have it examined when he or she wants to. That’s just a basic right. (ITC-U).

2. *Health checks should be performed in people likely to benefit most from the check*

In contrast, other providers state that people should not be free to participate in whatever health check, but that it would be best if health checks were performed in people likely to benefit most. To optimize the benefit-harm ratio of health checks, health checks should only be performed in people at high risk for disease. This will enhance the positive predictive value of the test, which depends on the prevalence of disease. Checks moreover should only be performed in those likely to implement health advice. An often-mentioned example is that checking for CVD is only useful when people are willing and capable to implement lifestyle changes that are required to lower risk:

Preventive research [including health checks YS] also has to do with what people are willing to do with the results (IC).

Only in people who are willing to take the consequences, testing [for CVD risk factors] is useful (ITC-U).

Some health check providers therefore advice to assess risk factors and likelihood of implementing health advice before the test and to perform health checks only in those people most likely to benefit from the test. They warn of commercial providers, but also of patient groups and medical specialists with a particular focus on the disease tested for, who may be:

very focused to detect so to speak the last patient with cancer left. Very driven to do so. Not surprising of course, for they are confronted with the consequences of cancer for people in their practice. (PSPO).

The downside of this focus would be that they may offer tests to people at low risk for disease, which may result in many false positives.

Benefits other than health, such as reassurance, may also be taken into consideration. According to the providers who state that health checks should only be performed in people who are likely to benefit most, people at low risk who are extremely worried should still be able to test, because reassuring them may improve their wellbeing although it’s unlikely health benefits will result:

If somebody is worried sick, and thinks ‘I’ve got it’ [lung cancer], than yes, that person should be given the possibility to check (PSPO).

3. *Follow up care of privately funded tests should not drain collective resources*

To ensure affordable health care, some providers caution about privately provided tests that may cause follow-up in regular health care possibly draining collective resources. The total body scan is often mentioned as an example:

It [a total body scan] produces a lot of unnecessary additional medical consumption. (…) Obstruction, unnecessary [follow up] research, that is a waste (...) Not just of money but of time. Time of the people who have to do those [follow up] tests, time in the hospital for things that are not useful, which makes other people having to wait longer (GP).

## Criteria in practice

So far the focus has been on criteria for good health checks according to providers. This final section is devoted to the role of these criteria in the current offers of health checks. Which checks do – according to providers – meet the criteria for good health checks, and which do not? Do providers themselves comply with criteria? Is it feasible to fulfil these criteria according to providers?

### Good’ and ‘bad’ health checks

During the interviews many providers had serious difficulties mentioning a health check that they considered to be ‘good’. Many of them mention other forms of prevention instead, such as vaccinations, easily accessible preventive activities such as sports and smoking cessation programs, and tackling the obesogenic environment:

Uhm, I’m in doubt.. I can not just name one, because you have indeed the false positives and treatment.. it isn’t always easy.. You’re talking about tests, not about preventive vaccinations, those kind of things, you’re really asking for a test (PSPO).

When asked for an example of a ‘bad’ check however, providers readily named tests, often the total body scan and tests on total cholesterol.

### Why providers don’t comply with the formulated criteria

Providers reflected on their own offer of tests to emphasise what they think is important in a test, and were asked whether their current practice leaves room for improvement and how this could be achieved. Providers readily admitted they didn’t consistently meet their own criteria for good checks. Some of them offer checks that may detect untreatable disease and tests with a low predictive value. They do not always sufficiently inform potential users, the follow up of some of checks drains on collective resources, and follow up care is not always provided. Especially help with lifestyle changes following checks on CVD risks is rare.

Several reasons were mentioned for this gap between theory and practice. First, many providers struggle with the tension between the importance they attach to reliable, valid health checks that improve health, and their belief that potential users – provided they are well-informed – should be free to choose any personal health check they want, including tests that are unreliable, invalid and are unlikely to improve health, for example because (potential) users do not belong to the target group:

I am a strong proponent of self-determination. If I want to have my creatinine checked, I believe this should be possible. And I also believe that if I want to have my PSA checked – which is such a discussion – I believe I should be able to have my PSA checked. And a GP may think I shouldn’t, but then I think this GP needs to explain to me why I shouldn’t. But even then I believe I should be able to request that [a PSA check]. If, of course, I fit into a specific group [population]. If I’d be 30, well.. No anyway, then it should still be possible (ITC-U).

Second, especially medical specialists express a strong desire to prevent serious illness and death from disease while (as yet) no test with good predictive value is available:

Look, a PSA test is obviously not a good test and should also not be used for screening. That’s not a good test, especially the specificity is much, much, much too low. But there is nothing better. (...) That is a real miserable death, prostate cancer is the most miserable death from cancer that you can imagine. You’ll get metastases in your bones, it will take three years, it is terribly painful, it’s an enormous decline because it is such a slow-growing killer. So look, and that’s exactly what I want to prevent, that misery (ITC-I).

Third, many providers, especially GPs, suffer from a lack of time. In practice they cannot properly inform, advise and assist (potential) users before and after the test:

I find it difficult in cases in which a patient says: I want my PSA checked, that just takes a moment isn’t it doctor? That happened a few times, to then explain as good as possible how it is (GP).

Fourth, although all respondents would like to provide good care, providers other than the government and GPs make (part of) their living selling health checks, so they have an interest that may conflict with offering reliable, valid checks that provide health benefits:

We are a commercial company as well... yeah. So I mean.. would you in such a situation also think “well, should I do this [offer this test] or not”? Yes that can be difficult, to make honest weightings, therein. Yes, I’m very honest about that (HSO).

A similar conflict is reported concerning the provision of objective and full information to potential users:

Really, I would.. I think you should give objective information. (...) Look the point is, and that has to do with market forces in health care, you’ll always .. the biggest .. uhm... challenge is in recruitment versus information. Recruitment meaning how do you recruit your clients, and how do you give the right information. (...) How do you do a.. recruitment in which you do alert people to potential risks or disadvantages (ITC-U).

### Informed consent may be difficult to establish

According to interviewed providers, informed consent is a minimal criterion. As just described, sometimes providers fail to supply potential users with sufficient information. A majority of the interviewed providers however questions whether true informed consent is feasible at all. Even if potential users would be provided with objective, reliable, valid, accessible and understandable information; this would not necessarily enable them to make informed assessments concerning benefits and harms of tests. This is because many people, according to providers, have difficulties with making ‘rational’ decisions in the context of health:

What people understand and what people want to understand, you can explain something very well, but sometimes people just want to read something else, so (ITC-U).

The context in which a check is offered is said to have a big influence on decisions (not) to test. When a test is free, easily accessible, if ‘everybody’ participates, and when potential users trust the provider, people would hardly consider information at all:

Yes, the more accessible it is, if it becomes common practice, the less people tend to think about what the consequences might be if you do that [testing] (PHA).

According to providers, health related choices are influenced by fear of death (rather safe than sorry). Faced with the possibility of serious disease that can be treated at an early stage, people are willing to accept unnecessary worry and even damage to health that may result from overdiagnosis and overtreatment. Many providers thus explain choices to test for prostate cancer.

People always tend to believe the worst case scenario. (…) A certain fear to have something, a fear of illness (…) [They take the PSA test] we’ll treat them and they will be glad we do. In the meantime they’re confronted with incontinence, impotence, that sort of thing. And surprisingly enough, they take it for granted. (...) They’ll reason I have prostate cancer and I’ve been treated .. so I escaped death (GP).

Interestingly, the very same providers also make comments in which nothing echoes the aforementioned nuances to the view of man as rational decision maker:

If they want to spent their money on that [total body scan YS] instead of on a vacation. Well, why not, that is their choice (GP).

People should just be smart enough to decide whether or not they participate in checks, ok? (ITC-U).

As long as information is provided, choices would simply reflect what people want. Providers make these comments to justify their own practice, but are also in relation to health checks offered by other providers.

## Discussion

### Providers’ criteria are demanding

When we compare the criteria formulated by the providers with existing criteria for personal health checks and population screening, five points stand out. First, the criteria as formulated by the interviewed providers resemble the criteria for responsible population screening closely. Analytic validity, clinical validity, clinical usefulness, benefits outweighing harms and informed consent are all considered minimum criteria. Providers also state that governmental screening should be cost-effective. Providers, thus, agree with ethicists that the Wilson and Jungner criteria are also applicable to personal health checks. [2, 4–6]

In the regulatory context of personal health checks, the opinions of providers however, give a new perspective. Their criteria are actually more demanding than criteria formulated for personal health checks thus far. Providers consider health benefits and clinical validity minimal criteria for personal health check, as for population screening. In contrast, clinical utility is mentioned in none of the existing criteria for personal health checks [1, 13–15] and clinical validity in only one. [1]

Third, the ideas about follow up care of interviewed providers are more demanding than criteria formulated for responsible population screening and criteria for personal health checks. In population screening people are informed about the meaning of results and referred to specialized care, criteria for personal health checks include ‘advice to clients on strategies the client can follow to reduce any further risk of acquiring a condition(s) or its negative consequences.’ ([13], see also [14]) Several interviewed providers however advise to *help* users in realizing the actions necessary to reduce risk.

Fourth, it is remarkable that interviewed providers make almost no remarks about privacy and storage of data. Criteria for responsible governmental screening state that offer and performance should be in accordance with patient and consumer rights. Criteria for personal health checks explicitly mention the handling of data. [1, 13–15] We think the fact that interviewed providers seem to take patient- and consumer rights as given could best explain this apparent omission (Additional file 3).

Last but not least, the interviewed providers have specific concerns about informed consent. Although they consider this a minimal criterion, they question its feasibility in practice. According to providers, people would be more influenced by context and emotions than by information in their decisions to test. This is a cause for concern, considering the importance placed on informed consent in the existing criteria for personal health check. In these criteria, informed consent is not only considered as a criterion in itself but also as a condition to ensure that individual benefits outweigh harms because informed individuals would be able to determine whether that is the case. [1, 13–15] According to interviewed providers this is, thus, not realistic. To ensure health checks provide more benefits than harms, they instead mention an individual assessment before the test, help with the implementation of health advice after the test, and providers being skilled and experienced professionals who put the benefit of (potential) users first and take time and attention in their provision of care. Although some proposals for the ethical evaluation of personal health checks mention assessment of risk factors [7, 9] or qualified providers [8, 9], their emphasis is on informed consent.

### Providers don’t meet criteria: Lessons

Interviewed providers have difficulties mentioning personal health checks that meet their criteria. Moreover, they do not consistently meet the formulated minimal and aspirational criteria in their own practice. Respondents name several reasons for this gap between theory and practice. We discuss their implications for the usability of criteria for personal health checks.

First, primarily the commercial providers struggle with the tension between the importance of reliable, valid checks that improve health and their belief that potential users should be free to choose any test they want, including unreliable, invalid tests that do not provide opportunities for health improvement, *provided they are well-informed.* This contested criterion (nr 1) is hard to reconcile with the minimal criteria, which these providers, thus, often fail to meet. There are commercial motives at play, but non-commercial providers also state that people at low risk that are extremely worried should be able to test, regardless the fact that the validity of test results will be negatively influenced and it is unlikely that health benefits will result. (see contested criterion 2) This apparent dilemma between avoidance of harm and respect for autonomy emphasises that a list of criteria may contain value conflicts that are not easily resolved.

Second, providers are sometimes faced with the dilemma that they want to prevent harm from disease, but have no options except health checks with poor predictive value, which may result in harm due to over-treatment and unnecessary worries. On a population level, it may not be easy to identify when health checks no longer provide more benefits than harms. On the level of an individual user or patient, providers – like individuals themselves – reason it is better to be safe than sorry.

These dilemma’s point out that in developing ethical criteria for offering health checks potential conflicts between criteria and underlying values should be explicated and addressed.

Other reasons why providers do not meet criteria point to components in the organisation of health care that may conflict with an ethical provision of health checks: Especially GPs report that they lack time to properly inform, advise and assist (potential) users before and after the test. This has been reported before in studies on preventive care in general practice. [23] They express moral frustration about this situation because they want to provide good care.

Some (semi) commercial providers have an interest that sometimes conflicts with offering reliable, valid checks that provide health benefits and giving objective and full information to potential users. Commercial providers are faced with the question why their practice should be moral if they could make money instead. The question ‘why be moral?’ may be even more central in the practice of the ‘small’ commercial providers without a (para) medical background which rejected our request for an interview by stating that this ‘would be at the expense of customers who pay € 225 per hour’.

These dilemmas point out that in thinking about the ethics of offering health checks; we should take the larger context into account. It is important to realize the conditions that would facilitate providers to meet the criteria. Other actors, like the government or insurance companies, need to take responsibility here as well.

When it comes to the usability of general criteria for personal health checks there is of course also the general problem of the application of criteria. As mentioned in the introduction, the criteria for population screening may be relatively uncontested, but whether or not specific population screening programs meet these criteria is subject of debate. [9] Agreement about general criteria for personal health checks is thus only a first step towards the ethical evaluation of specific checks. When is a test ‘reliable’ and ‘valid’ enough? General criteria must be interpreted for specific tests, and it may well be that different providers will interpret criteria differently or that there will be dissensus when it comes to applying general criteria for specific tests. To our mind, standards should be at least as demanding as those of population screening.

### Contested criteria: Ethical evaluation

The criteria as formulated by providers should not be taken as a final say, but used as input in the ethical and policy debates over the right criteria. Their minimal and aspirational criteria are in line with what most ethicists consider to be good (personal) health check offers. [2, 4–6] This is different however, when it comes to contested criteria. In this section we provide an ethical evaluation of these contested criteria and explore their relevance for an overarching ethical framework for responsible offer and use of personal health checks.

#### Respecting autonomous choice

The idea that people should be free to participate in unreliable, invalid checks on (risk-factors for) disease that cannot be treated *provided that they are well-informed* may be seen as an attempt to prevent harm from checks while respecting autonomous choice. Autonomy is a key value in medical ethics. [24] We briefly discuss how we understand autonomous choices in the context of health checks and evaluate this criterion on this basis.

An autonomous choice is a choice made in freedom, a choice that is in line with somebody’s values, with his or her perception of the good life, a choice that ideally is based on reliable information. [2, 24] Interestingly, many providers – commercial and non-commercial – think people would be more influenced by context and emotions than by information in their decisions to test. Indeed people may not be capable of using the information provided during informed consent procedures in decisions to test because they lack the health literacy to do so [25, 26] or because emotions or heuristics override rational considerations. [27].^8^

If providing information to potential users does not necessarily lead to autonomous choices (not) to participate in health checks, how to evaluate these choices? This should depend on whether that choice is conductive to this individuals’ own perception of a good life. [2] Most people value health more than anything else. [28] In addition, a choice for a test should be in line with what the individual wants or expects from the check. [29] Many people expect tests to have a good predictive value, to offer opportunities for health improvement and not to inflict any harm [30] and participate for reasons of health. (e.g. [31]).

From this we may conclude that offering tests with a low predictive value that do not provide health benefits is not likely to be conductive to the autonomy of (potential) users. This is because most people value health, expect health check to provide certainty about the presence or absence of disease and test to improve health. [29–31] Moreover, providing information about test- and disease characteristics that are contrary to what people expect will probably not suffice to prevent harm because decisions (not) to test are more often made on the basis of emotions and heuristics than information. [25–27] The idea that people should be free to participate in whatever check, *provided that they are well-informed* is thus not likely to result in respect for autonomous choice while preventing harm from checks.

This is different when potential users would be ‘informed’ in individual assessments, as some providers suggest in the context of contested criterion 2. In such assessments, risk factors and likelihood of health advice being implemented would be discussed and the individual needs of potential users would be deliberated upon. [32] This way, unrealistic expectations may be corrected [29] and individual benefits and harms could be determined in the context of individual needs. Such individual assessments – perhaps best comparable to counselling in clinical genetics – would require personal contact and thus take time and attention from providers. They may also require offering less comprehensive tests. For example, it does not seem realistic to discuss all relevant benefits and harms of a total body scan or broad genome-wide testing. [33]

In case of health checks with a low predictive value or checks that do not provide opportunities for health improvement, we thus suggest performing individual assessments as ‘alternative’ informed consent procedures. This way, it is assured that participating is in line with individual’s values and expectations and provides more benefits than harms.

This analysis of autonomous choice and the limitations of informed consent has implications beyond an ethical provision of health checks. In general we think it is important to design informed consent procedures such that they truly function as means to respect the autonomy of (potential) users. [24]

#### Taking the context into account

The suggestion of some providers to perform in particular cardiovascular health checks only in people who are willing and capable to implement any lifestyle advice could, like a recent, very similar advice from the US Preventive Service Task Force [34], be criticized as ‘withholding potential benefit from the population subgroups whose socioeconomic burdens and comorbidities place them in greatest need of help’. [35]. After all, the low SES groups who are, according to interviewed providers, not likely to implement health advice have on average a bigger risk on cardiovascular disease. [36, 37]

However, a different picture arises if we would evaluate this suggestion from a ethical perspective in which the context is taken into account. We think that a true ethical provision of health checks requires weighing benefits and harms of health checks against other measures targeting (risk factors for) disease. Let us weigh the benefits and harms of cardiovascular health checks up to benefits and harms of other measures targeting (risk factors for) CVD: asking people to change their lifestyle if they’re not capable to, is not likely to improve their health but may cause worries and feelings of guilt. [3] Low SES groups indeed deserve care, but may need different care than the provision of cardiovascular health checks. [31] It would be more fair and effective to help those people maintaining a healthy lifestyle by changing the obesogenic environment they live in. [38–40] Thinking about the ethics of health checks may thus also result in an advice to implement other measures than health checks, for certain subgroups or in general. To change the obesogenic environment other actors than providers need to be addressed, such as governments and industry. [3]

### Limitations of the study

We have chosen a qualitative approach because we are the first to question providers on an ethical provision of health checks and the openness of a qualitative method allows the uncovering of unexpected views. The aim was an in-depth understanding of what providers consider criteria for good personal health checks and potential barriers in the implementation of these.

Although we interviewed a very divers sample of providers, saturation as regards to criteria was reached long before the last interview: they formulated very similar and ‘demanding’ general criteria. We did not expect this to happen. Despite differences in education(al) level, profession, work field and interest, providers – apparently – have very similar ideas when it comes to the ethics of health checks. Although we cannot exclude the possibility of a social acceptability bias in their answers – respondents were aware that they were interviewed by an ethicist and perhaps did not want to come across as thoughtless – however we do not consider this a likely scenario. The atmosphere during most^9^ interviews was perceived by YS and described by respondents as surprisingly open and relaxed.

In addition, providers were aware that their answers would be employed in the development of an ethical framework for personal health checks. Strategically providers had every reason to be somewhat less demanding while showing goodwill to make some adjustments in their offers. Such a position after all would minimize the chances of (governmental) involvement in their practices. That being said, our results may suffer from a sampling bias: ‘Small’ commercial providers without a (para) medical background who declined our interview requests would perhaps have formulated less ‘demanding’ criteria. And the two providers of governmental screening programs came up with criteria very similar to criteria for responsible governmental screening, as was perhaps to be expected.

To draw conclusions on how many providers value which criteria for good health checks, the results of this study would need to be quantified.

A potential shortcoming of this study is the relatively short section on criteria in practice as these results are the most interesting from an ethical point of view. The reason why we do not have more material to present is that we wanted providers to be as open and honest about their practices as possible in an interview with an ethicist, and figured they would be more at ease if we did not question them too directly and long about whether and why they (do not) keep to criteria.

## Conclusions

Providers are more demanding than existing ethical criteria for personal health checks and state that personal health checks should meet the following minimum criteria: (Risk factors for) disease to which the check is directed should be treatable; Tests must be reliable and clinically valid; Participation must be informed and voluntary; Health checks should provide more benefits than harms; Governmental screenings should be cost-effective. Some providers also formulate aspirational criteria: Follow-up care should be provided; Providers should be skilled and experienced professionals that put the benefit of (potential) users first; Providers should take time and attention. Providers disagreed over the following contested criteria: People should be free to test on any (risk factor for) disease; Health checks should only be performed in people at high risk for disease that are likely to implement health advice; Follow up care of privately funded tests should not drain on collective resources.

Providers do not always fulfil their own criteria. Their reasons reveal conflicts within the set of criteria for good checks, conflicts between criteria and other ethical values, and point to components in the organisation of health care that hinder an ethical provision of personal health checks. Moreover, many interviewed providers consider informed consent a criterion that is hard to establish in practice. An individual assessment in which risk factors, the likelihood of health advice being implemented and the needs of potential users would be discussed is an interesting suggestion for an informed consent procedure that truly functions as means to respect the autonomy of (potential) users. [24]

The results of this study suggest that in thinking about the ethics of health checks, drawing up a checklist of criteria will not suffice. Potential conflicts between criteria and their values should be explicated and preferably help in the weighing of criteria should be provided. Moreover, in the ethics of health checks it is important to take the larger context into account. It is important to realize the conditions that would facilitate providers to meet the criteria. Actors, like the government or insurance companies, need to take responsibility here as well and thus, should be addressed. Moreover, an ethical framework for health checks should enable weighing up benefits and harms of health checks to other measures targeting (riskfactors for) disease. An ethical evaluation of health checks within this larger context may result in an advice to implement or promote other measures than health checks.

## Additional files

Additional file 1: Interview guide (DOCX 126 kb)

Additional file 2: Profession and age of health check providers (DOCX 64 kb)

Additional file 3: Comparison of criteria. Comparison between criteria as mentioned by interviewed providers and what is written on these criteria (eg what is mentioned about informed consent) in criteria for responsible population screening and existing criteria for personal health checks (DOCX 114 kb)

## Abbreviations

CVD: Cardiovascular disease
EA: Eva Asscher
GP: General Practitioner
GPSPO: Screening at GP’s Program Officer
HCN: Health Council of the Netherlands
HSO: Health and Safety Officer
IC: Internet based company
ITC-I: Independent Treatment Centre – Insured care
ITC-U: Independent Treatment Centre – Uninsured care
MS: Maartje Schermer
PHA: Pharmacist
PHY: Physiotherapist
PSA: Prostate specific antigen
PSPO: Population Screening Program Officer
STD: Sexually transmittable diseases
YS: Yrrah Stol

## Footnotes

1 The screening and any subsequent interventions should lead to an improved health outcome among people with a positive test result.

2 The accuracy with which the test measures a specific biomarker.

3 The accuracy with which the test identifies a particular (risk factor for) disease.

4 Note that many more guidelines for direct to consumer (DTC) genetic tests exist. [41, 42] We however, only discuss guidelines applicable to all sorts of personal health checks.

5 In the Netherlands, people need a referral from their GP to be treated or examined by a medical specialist in regular/insured care. In contrast, care by specialists in commercial/uninsured care is accessible without GP referral. Specialized medical care may either be provided in hospitals (regular care) or in independent treatment centres (either insured or uninsured care).

6 There is currently much public debate in the Netherlands concerning the Total Bodyscan. Thus far, performance is forbidden in the Netherlands. However, it is offered and performed just outside Dutch borders. This may have influenced how often providers refer to this test during the interviews.

7 The Netherlands has a special law on screening: the Population Screening Act. As explained in the introduction it states that population screening must benefit participants. It operationalizes this through a mandatory permit for (expected) harmful screening: that uses ionizing radiation, aims to detect cancer or diseases for which no treatment or prevention. Grounds to refuse the permit are 1. The screening test is scientifically unsound; 2. The screening is not conform the legal rules for medical treatment; 3. The expected benefit of the screening does not outweigh the risks for the participants. To determine the risk-benefit ratio for participants, the Wilson and Jungner criteria are used. (HCN 2008) During the time of the interviews, the minister of health of the Netherlands deliberated on possible adjustment of the Population Screening Act.

8 One may wonder why providers state that people should be free to test provided they are well informed, if they think people would be more influenced by context and emotions than by information in their decisions to test. As discussed the very same providers that question informed consent also make rather indiscriminate statements about self-determination: choices would simply reflect what people want. As most providers came across as genuinely committed to the well-being of the users of their test, a mechanism called ‘cognitive dissonance’ may be at play. [43] Cognitive dissonance refers to the psychological stress that results from holding a belief that is contradictory to ones actions. To reduce stress, people may either adapt their ideas or their actions. [43] To fit their practice in which they perform unreliable, invalid health checks that do not always provide opportunities to improve health but may even harm health, providers may simplify their conception of self-determination. Another or additional explanation as to why providers state people should be free to test provided they are well informed is that they may hold on to informed consent procedures as if it were an ethical anchor. Informed consent procedures as something intrinsically good would then outweigh uncertain harms done by unreliable or invalid checks that do not provide opportunities for health improvement. Although this is an understandable thought given the attention within medical ethics to informed consent [24], informed consent should be understood as a means to respect the autonomy of (potential) users, not an end in itself.

9 With the exception of two interviewees who seemed on their guard.
