# Supplementary material for: What is a good health check? An interview study of health check providers’ views and practices
Source: BMC Med Ethics. 2017 Oct 2;18:55. doi: 10.1186/s12910-017-0213-x (PMC5625608; doi:10.1186/s12910-017-0213-x)
Supplement: Supplementary file 1 — Interview guide (DOCX 126 kb) [file 12910_2017_213_MOESM1_ESM.docx]

**Interview guide**

Could you please tell us something about your daily practice? What kind of health checks do you offer? Who are you users? Why do you think they test? How do they react on results?

Who pays for the health check?

What are the benefits of testing of your test(s)? What do you offer people?

How do you offer you health checks? What information do you provide (potential) users with?

Are there disadvantages of your test(s)? If yes, which ones? How do you think about that?

Could you name an example of a really good health check?

What makes this check such a good check? How is it different from other checks?

To which group of potential users do you think this check should best be provided?

Which provider would be suited best to offer this check, and how should this check be provided?

Could you, in general, name characteristics of good health checks?

Could you also name an example of a health check you consider to be really ‘bad’?

What makes this test such a bad check? How is it different from other checks?

Would it make any difference which provider would offer this check in which way and to whom? Why?

Do you think this test should be prohibited, or maybe warned for? Why (not)?

Could you, in in general, name characteristics of bad health checks?

Do you consider the following check ‘good’, ‘bad’ or something in between, and for what reason? 1. Cardiovascular health checks; 2. PSA tests; 3. A total body scan; 4. Mammography; 5. Stool test; 6. Cervical smear; 7. Genetic tests (in random order).

I’ll name different kind of health checks and I want to ask you to think of a really good person, somebody you admire for his or her goodness. What would the practice of this person look like, if he or she would offer health checks?

If that person would ‘run’ your practice, would he or she do anything different? If yes: what would he or she change and for what reason?

Why don’t you act just like this person? Could you identify barriers between your practice and that of this person who embodies goodness?

Could the government be of any help in the removal of these barriers between you practice and the practice of a person who embodies goodness? How?

Still striving for the ‘good’, do you think it would make any difference what kind of providers offer health checks? Which providers could best offer them, and how?

Could the government take this into account? How?

If you would be asked to give the government advice on the governance of health checks, what would you say?
