# Supplementary material for: What is a good health check? An interview study of health check providers’ views and practices
Source: BMC Med Ethics. 2017 Oct 2;18:55. doi: 10.1186/s12910-017-0213-x (PMC5625608; doi:10.1186/s12910-017-0213-x)
Supplement: Supplementary file 2 — Profession and age of health check providers (DOCX 64 kb) [file 12910_2017_213_MOESM2_ESM.docx]

**Profession and age of providers**

|  | **Profession/role** | **Approximate age** |
| --- | --- | --- |
| 1 | General Practitioner | 60 |
| 2 | General Practitioner | 50 |
| 3 | General Practitioner | 50 |
| 4 | General Practitioner | 65 |
| 5 | Key player GP screening program | 45 |
| 6 | Key player screening program | 50 |
| 7 | Key player governmental screening program | 40 |
| 8 | Physiotherapist | 30 |
| 9 | Physiotherapist | 55 |
| 10 | Director of health and safety service | 60 |
| 11 | Health and safety officer | 45 |
| 12 | Key player pharmacy chain | 55 |
| 13 | Key player pharmacy chain | 50 |
| 14 | Founder & medical specialist independent treatment centre – insured care | 65 |
| 15 | Founder & medical specialist independent treatment centre – uninsured care | 45 |
| 16 | Founder independent treatment centre – uninsured care | 40 |
| 17 | Key player & medical specialist independent treatment centre – uninsured care | 60 |
| 18 | Key player & medical specialist independent treatment centre – uninsured care | 50 |
| 19 | Founder & medical specialist internet based commercial company | 55 |
| 20 | Key player internet based commercial company | 40 |
