# Supplementary material for: What is a good health check? An interview study of health check providers’ views and practices
Source: BMC Med Ethics. 2017 Oct 2;18:55. doi: 10.1186/s12910-017-0213-x (PMC5625608; doi:10.1186/s12910-017-0213-x)
Supplement: Supplementary file 3 — Comparison of criteria. Comparison between criteria as mentioned by interviewed providers and what is written on these criteria (eg what is mentioned about informed consent) in criteria for responsible population screening and existing criteria for personal health checks (DOCX 114 kb) [file 12910_2017_213_MOESM3_ESM.docx]

| **Criterion formulated by interviewed providers** | **Criteria**  **for population screening ([5]**  **summary)** | **Existing criteria for personal health checks** | | | |
| --- | --- | --- | --- | --- | --- |
|  |  | **European quality criteria [7]** | **Human Genome Commission [8]** | **Health Council of the Netherlands [1]** | **Guideline Royal Dutch Medical Association [9]** |
| Health checks must provide clear opportunities for health improvement | The usefulness of detecting a given disease (or its associated risk factors) must be clearly established |  |  |  |  |
| The health check must be reliable and valid | Test method involved should be reliabe and valid |  |  | [7] Suitability of a test must be demonstrated by research on clinical validity. |  |
| Participation voluntary and based on reliable information | Participation voluntary and based on reliable information | [3.1] [3.2] [3.4] and [3.5] on information provision and explicit informed consent | [4.1] [4.3] [4.4] and [6.2] on information provision and free and informed consent | [6] [7] and [12] on information provision and voluntary and informed consent. | [2.2.1] 2.1.4] and [2.2.1] on information provision and explicit informed  consent |
| Health checks should provide more benefits than harms for the individual | Benefits involved must outweigh the drawbacks for the individual |  |  | [12] Advantages should outweigh disadvantages for participants. | [1.2.4] The benefits of the test for the target group should outweigh the adverse effects or risks. |
| Cost-effective in case of governmental screening programs | The screening must be responsible, in terms of the use it makes of public and collective health care resources |  |  |  |  |
| Explanation test results and follow up care  Help with realization treatment/  lifestyle adjustment | Treatment should lead to a better prognosis | [3.6 and [3.7] on explanation test results and follow up | [4.9] [4.10] [11.1] and [12.1] on explanation test results and follow up | [8] on explanation test results and follow up | [2.5.1] [2.5.2] [2.5.3] [2.6.2] and [3.6] on explanation test results and follow up |
| Skilled and professional provider |  |  | [5.2] [9.4] and [10.1] on qualifications counsellor, laboratory personnel and professional responsible for interpretation test results |  | [3.3.1] on qualifications employees involved in implementation of a test |
| Provider taking time and attention |  |  |  |  |  |
| Freedom to test |  |  |  |  |  |
| Risk factors, motives and likelihood of implementing health advice should be assessed before the test. | Screening is only available for high risk groups | [3.3] on risk assessment to ascertain whether user belongs to the target population. |  |  | [2.1.2] and [2.2.2] user belongs to the target group of the test. If not providers advise not to test. |
| Follow up care of privately funded tests should not drain collective resources |  |  |  |  |  |
|  | In accordance  with patient rights (if offered outside the health care system: consumer rights) | [3.8] quality-, client/patient safety- and information security management, and storage and handling of residual material in accordance with National/European/ International standards | Principles should be used in accordance with applicable international instruments and domestic law. [2.1] [7.1] and [8.1] on advertising, privacy, storage and handling of biological samples | [9] on handling and storing medical data, privacy of participants | [3.2.3] in accordance with applicable laws and regulations; relevant industry guidelines |

Summary of what is written on each criterion (eg what is mentioned about informed consent) in criteria for responsible population screening and existing criteria for personal health checks
